# Polymorphisms in methylenetetrahydrofolate reductase and cystathionine beta-synthase in oral cancer – a case–control study in southeastern Brazilians^☆^

**DOI:** 10.1016/j.bjorl.2015.10.012

**Published:** 2015-12-17

**Authors:** Andressa Barbosa, Marcelo dos Santos, José Roberto Vasconcelos de Podestá, Sônia Alves Gouvêa, Sandra Ventorin Von Zeidler, Iúri Drumond Louro, Melissa de Freitas Cordeiro-Silva

**Affiliations:** a Universidade Federal do Espírito Santo (UFES), Departamento de Ciências Biológicas, Vitória, ES, Brazil; b Hospital Santa Rita de Cássia, Divisão de Cirurgia de Cabeça e Pescoço, Programa de Prevenção e Detecção Precoce de Câncer Oral, Vitória, ES, Brazil; c Universidade Federal do Espírito Santo (UFES), Departamento de Ciências Fisiológicas, Vitória, ES, Brazil; d Universidade Federal do Espírito Santo (UFES), Departamento de Patologia, Vitória, ES, Brazil; e Faculdade Católica Salesiana do Espírito Santo, Vitória, ES, Brazil

**Keywords:** Oral squamous cell carcinoma, Methylenetetrahydrofolate reductase, Cystathionine beta-synthase, Genetic polymorphism, Carcinoma epidermoide oral, Metilenotetrahidrofolato redutase, Cistationina beta-sintase, Polimorfismo genético

## Abstract

**Introduction:**

Oral squamous cell carcinoma (OSCC) is a serious public health problem, due to its high mortality rate and worldwide rising incidence. OSCC susceptibility is mediated by interactions between genetic and environmental factors. Studies suggest that genetic variants encoding enzymes involved in folate metabolism may modulate OSCC risk by altering DNA synthesis/repair and methylation process.

**Objective:**

The goals of this study were to evaluate the association of three genotypic polymorphism (*MTHFR* C677T, *MTHFR* A1298C and *CBS* 844ins68) and oral cancer risk in southeastern Brazilians and evaluate the interactions between polymorphisms and clinical histopathological parameters.

**Methods:**

This case–control study included 101 cases and 102 controls in the state of Espírito Santo, Brazil. *MTHFR* genotyping was done by PCR-RFLP (polymerase chain reaction – restriction fragment length polymorphism) and *CBS* genotyping by PCR (polymerase chain reaction) analysis.

**Results:**

*MTHFR* C677T polymorphism was associated with lymph node involvement. Genotype CT + TT acted as a protective factor. *MTHFR* A1298C AC + CC genotype was associated with tumor differentiation, and possibly with a better prognosis. In risk analysis, no correlation was observed between genotypes and OSCC.

**Conclusion:**

We concluded that *MTHFR* C677T, *MTHFR* A1298C and *CBS* 844ins68 polymorphisms were not associated with OSCC risk in southeastern Brazilians; however, we suggest a prognosis effect associated with *MTHFR* C677T and A1298C polymorphisms in OSCC.

## Resumo

**Introdução:**

O carcinoma espinocelular oral (CECO) trata-se de um importante problema de saúde pública, devido à elevada taxa de mortalidade e incidência crescente em todo o mundo. A susceptibilidade ao CECO é mediada por interações entre fatores genéticos e ambientais. Estudos sugerem que as variantes genéticas que codificam as enzimas envolvidas no metabolismo do folato podem modular o risco de CECO, alterando a síntese/reparação do DNA e o processo de metilação.

**Objetivo:**

Os objetivos deste estudo foram avaliar a associação de três polimorfismos genotípicos (*MTHFR* C677T, *MTHFR* A1298C e *CBS* 844ins68) e o risco de câncer bucal em brasileiros da região Sudeste, e avaliar as interações entre polimorfismos e parâmetros clínico-histopatológicos.

**Método:**

Este estudo de caso-controle incluiu 101 casos e 102 controles no estado do Espírito Santo, Brasil. A genotipagem do polimorfismo *MTHFR* foi realizada por PCR-RFLP (Reação de Polimerase em Cadeia – Polimorfismo no Comprimento de Fragmento de Restrição) e a do *CBS* por análise da PCR (Reação de Polimerase em Cadeia).

**Resultados:**

O polimorfismo *MTHFR* C677T foi associado ao envolvimento de gânglios linfáticos. O genótipo CT + TT atuou como um fator protetor. O genótipo *MTHFR* A1298C AC + CC foi associado à diferenciação do tumor e, possivelmente, a um prognóstico melhor. Na análise de risco, a correlação entre os genótipos e o CECO não foi observada.

**Conclusão:**

Concluímos que os polimorfismos *MTHFR* C677T, *MTHFR* A1298C e *CBS* 844ins68 não estão associados ao risco de CECO nos brasileiros da região Sudeste; no entanto, sugerimos um efeito prognóstico associado aos polimorfismos *MTHFR* C677T e A1298C em CECO.

## Introduction

Oral squamous cell carcinoma (OSCC) is the eighth most common human cancer worldwide.^1^ In Brazil, nearly 15,290 new cases of oral cancer are expected in 2014, and in the Southeast it is the fourth among men and tenth among women.^2^ OSCC is a multifactorial disease, affected by notorious environmental factors such as alcohol and tobacco, as well as genetic factors, of which little is known. Polymorphisms in certain genes may confer susceptibility to OSCC development. Studies have shown a relationship between polymorphisms of genes involved in folate metabolism and OSCC risk due to their influence on methylation, synthesis and DNA repair.^3^, ^4^, ^5^, ^6^, ^7^

*MTHFR* gene encodes the methylenetetrahydrofolate reductase enzyme, that is important for intracellular folate homeostasis and the irreversible conversion of 5,10-methylenetetrahydrofolate (5,10-MTHF) into 5-methyltetrahydrofolate (5-MTHF). Polymorphisms C677T and A1298C in the *MTHFR* gene may be associated with oral cancer susceptibility due to changes in catalytic activity. The C677T polymorphism results in an enzyme with 65 percent of the wild-type homozygote activity for heterozygotes and 30 percent for homozygotes of the variant allele.^8^, ^9^ The *MTHFR* A1298C polymorphism is localized in the regulatory domain region.^10^ Homozygous 1298C individuals have approximately the same enzyme activity of those who are heterozygous.^11^ Reduced MTHFR enzyme activity increases the availability of folate for the production of thymidylate and purine for DNA synthesis and repair.^12^

The *CBS* gene encodes cystathionine beta synthase (*CBS*), also involved in the folate pathway, which mediates the conversion of homocysteine to cystathionine. *CBS* 844ins68 polymorphism has been associated with shorter survival time in head and neck squamous cell carcinoma patients.^13^ Therefore, this study aimed to investigate the frequency and association of *MTHFR* and *CBS* polymorphisms in oral cancer susceptibility in the population of the state of Espírito Santo, Brazil and its potential impact on the prognostic outcome.

## Methods

### Samples

In this case–control study, blood samples were collected from 101 patients with conclusive histopathological diagnostic of oral squamous cell carcinoma obtained from the Head and Neck Division of the Hospital Santa Rita de Cássia, Brazil. Of this total, 69 individuals were classified by skin color and other physical traits: 22 as white (Caucasians, mainly Portuguese descendents); 32 as “pardos” (ethnic mixture of Europeans, Africans and Amerindians), and 15 as black (African descendants), based on the official Brazilian census categorization. All patients were residents of the state Espírito Santo (ES, Brazil) and randomly recruited from 2011 to 2013. The inclusion criteria during this study were patients of both genders, over 35 years of age who accepted to participate in the study. The exclusion criteria were patients with SCC in other sites and those who received radiotherapy, chemotherapy, surgery or any other treatment prior to recruitment.

The control group was composed by 102 individuals residing in ES, Brazil, who were referred for clinical assessment and had a negative cancer familial history and did not show pre-malignant or malignant oral lesions at the time of sample collection. The control group was matched by age and gender. As only partial data was available for the controls regarding the habits such smoking and alcohol usage, it was not included for matching with the cases. All subjects provided signed informed consents approved by institutional review boards. This work was previously approved by the local research ethics committee (CEP Protocol n° 318/2011).

Clinicopathological features of patients analyzed were tumor stage (early stage I–II and late stage III–IV), size (T1, T2, T3 and T4), nodal status (positive N+ and negative N0) according to TNM Classification,^14^ histolopathological grade (well, moderately and poorly differentiated tumors)^15^ and smoking habit. All required information about clinical and histopathological parameters was obtained from medical records. Participants were not classified into ethnic groups or skin color.

### Genotyping assays

Genomic DNA was isolated by phenol-chloroform extraction. The *MTHFR* C677T and *MTHFR* A1298C polymorphisms were genotyped by the PCR-RFLP (polymerase chain reaction – restriction fragment length polymorphism) method, as previously described.^8^, ^11^ All reactions included positive and negative controls. Approximately 20 percent of the samples were randomly selected to repeat the genotyping procedure. The reproducibility was 100 percent.

The C → T transition creates a restriction site for the enzyme *Hinf* I. PCR product (198 bp) was digested using Hinf1 and visualized by electrophoresis in 8 percent polyacrylamide gels and silver nitrate staining. PCR products included a single 198 bp fragment for wild-type homozygotes (CC); 198 bp, 175 bp and 23 bp fragments for heterozygote (CT), and 175 and 23 bp for mutant homozygote (TT).

The *MTHFR* 1298AC polymorphism eliminates the MboII restriction site.

Wild genotype (AA) produced five fragments of 56, 31, 30, 28 and 18 bp, whereas heterozygous (AC) yielded six fragments of 84, 56, 31, 30, 28 and 18 bp, and homozygous mutants (CC) produced four fragments of 84, 31, 30 and 18 bp.

*CBS* 844ins68 polymorphisms were characterized by differential size separation after PCR, as previously described.^16^ The polymorphic allele results from the insertion of 68 bp at exon 8. The major allele (I) presented a 239 bp fragment, and the normal allele (N) presented a 171 bp fragment.

### Statistical analysis

Genotypic frequencies were tested for Hardy–Weinberg equilibrium (HWE). The chi-square and Fisher exact tests were used for association analysis, and confirmation was obtained by the Lilliefors test (significance considered when *p* < 0.05). Multivariate logistic regression was used to obtain odds ratio (OR) and confidence intervals (95% CI). Statistical calculations were performed using the Epi InfoH v 3.4.3, 2007 software. Linkage disequilibrium (LD) and halotype analysis were conducted by Haploview software.

## Results

Characteristics of 101 oral cancer patients and 102 controls are shown in Table 1. Significant differences between groups were not observed (*p* > 0.05).

**Table 1:** Clinical characteristics of cancer patients and control subjects.

Characteristics	Patients	Controls	*p*-value
	*n* (%)	*n* (%)	
*Gender*			
Female	20 (19.8)	20 (19.6)	0.972
Male	81 (80.2)	82 (80.4)	

*Age, years*			
≤55	47 (46.5)	56 (54.9)	0.233
>55	54 (53.5)	46 (45.1)	

*Ethnic group*			
Whites	22 (31.9)	–	–
“Pardos” (mixed race)	32 (46.4)	–	
Blacks	15 (21.7)	–	

*Tobacco exposure*			
Consumer	73 (72.3)	0 (0.0)	–
Non-consumer	28 (27.7)	0 (0.0)	
Unknown^a^	0 (0.0)	102 (100.0)	

*Tumor stage*			
Early stage (I, II)	25 (24.7)	–	–
Advanced (III, IV)	76 (75.3)	–	

*Tumor size*^c^			
T1	12 (11.9)	–	–
T2	22 (21.8)	–	
T3	17 (16.8)	–	
T4	50 (49.5)	–	

*Nodal status*^c^			
N0	50 (49.5)	–	–
N+	51 (50.5)	–	

*Histopathological grade*			
Well	25 (24.7)	–	–
Moderately	29 (28.7)	–	
Poorly	5 (5.0)	–	
Not available^b^	42 (41.6)	–	
*Total*	101 (49.8)	102 (50.2)	

a Unknown (not considered in the statistical calculations).

b Not available (not considered in the statistical calculations).

c TNM classification.

Genotype frequencies for *MTHFR* C677T, A1298C and *CBS* 844ins68 in controls and oral cancer patients are shown in Table 2.

**Table 2:** Distribution of *MTHFR* and *CBS* genotypes among oral cancer patients and control groups.

Genotypes	Total	Patients	Controls	*p*-value
	*n* (%)	*n* (%)	*n* (%)	
*MTHFR* C677T				
CC	100 (49.2)	50 (49.5)	50 (49.0)	0.438
CT	86 (42.4)	45 (44.6)	41 (40.2)	
TT	17 (8.4)	6 (5.9)	11 (10.8)	

*MTHFR* A1298C				
AA	113 (55.7)	60 (59.4)	53 (52.0)	0.541
AC	80 (39.4)	36 (35.6)	44 (43.1)	
CC	10 (4.9)	5 (5.0)	5 (4.9)	

*CBS* 844ins68				
NN	163 (80.3)	76 (75.2)	87 (85.3)	0.112
NI	38 (18.7)	24 (23.8)	14 (13.7)	
II	2 (1.0)	1	1	
Total	203 (100.0)	101 (49.8)	102 (50.2)	

N, non-insertion; I, insertion.

Genotype distribution of the three genetic polymorphisms was not significantly different between oral cancer and control group (*p* > 0.05) (Table 2). *MTHFR* C677T, A1298C and *CBS* 844ins68 allele frequencies in controls and oral cancer patients are shown in Table 3. All tested polymorphisms are in the HWE.

**Table 3:** Distribution of *MTHFR* and *CBS* alleles among the oral cancer patients and control groups.

Allele	Patients			Controls			*p*-value
	*n* (%)	HWE		*n* (%)	HWE		
		*χ*^2^	*p*-value		*χ*^2^	*p*-value	
*MTHFR* C677T							
C	145 (71.8)	1.006	0.316	141 (69.1)	0.348	0.555	0.556
T	57 (28.2)			63 (30.9)			

*MTHFR* A1298C							
A	156 (77.2)	0.018	0.893	150 (73.5)	1.193	0.275	0.387
C	46 (22.8)			54 (26.5)			

*CBS* 844ins68							
N	176 (87.1)	0.357	0.550	188 (92.2)	0.260	0.610	0.096
I	26 (12.9)			16 (7.8)			

HWE, Hardy–Weinberg equilibrium; *χ*^2^, chi-square; N, non-insertion; I, insertion.

Haplo View software was used for conducted LD and haplotype analysis on alleles of *MTHFR* C677T and A1298C polymorphisms (Table 4). We found that there was no difference for the haplotypes in the two groups (*p* > 0.05), which suggested that the haplotypes do not increase the risk of cancer.

**Table 4:** Linkage disequilibrium and haplotype analysis for alleles of C677T and A1298C polymorphisms.

Haplotype	Cases 2*n* = 202	Controls 2*n* = 204	*χ*^2^	*p*-value	OR (95% CI)
C-A	301	291	–	–	1
C-C	191	195	0.173	0.676	0.946 (0.817–1.364)
T-A	231	213	0.141	0.706	1.048 (0.745–1.220)
T-C	103	117	1.040	0.307	0.851 (0.861–1.602)

Patients were evaluated by ethnicity, but a prevalence of polymorphic allele in ethnic groups was not observed. The prevalence of the C allele (*MTHFR C677T* polymorphism) was higher in the three ethnic groups (white – 63.6 percent; black – 73.3 percent, and “pardos” – 82.8 percent). For the *MTHFR* A1298C polymorphism, the A allele predominated in all ethnic groups (white – 72.7 percent; black – 83.3 percent, and “pardos” – 76.6 percent), and finally, the presence of the wild-type allele for CBS 844ins68 polymorphism was also more prevalent in all ethnic groups (white – 86.4 percent; black – 86.7 percent, and “pardos” – 89.1 percent). These data demonstrate the ample mixture of races in the case group.

Interaction between genotypes and clinicopathological features was further analyzed (Table 5, Table 6). *MTHFR* C677T polymorphism is associated with positive lymph nodes, and the combination of CT + TT genotypes acts as a protective factor. Multivariate analysis (95% CI) considering tumor size revealed that the combination of CT + TT genotypes generates a risk of lymph node metastasis three times smaller than the CC genotype (*p* = 0.012) (Table 7).

**Table 5:** Clinicopathological characteristics of patients with OSCC and relation with the *MTHFR* polymorphisms studied.

Features	*MTHFR* C677T genotype					
	CC	CT	TT	*p*-value	CT + TT	*p*-value
	*n* (%)	*n* (%)	*n* (%)		*n* (%)	
*Tumor stage*						
Early stage (I, II)	12 (24.0)	11 (24.4)	2 (33.3)	0.880	13 (25.5)	0.862
Advanced (III, IV)	38 (76.0)	34 (75.6)	4 (66.7)		38 (74.5)	

*Tumor size*^a^						
T1	5 (10.0)	5 (11.1)	2 (33.3)	0.569	7 (13.7)	0.716
T2	12 (24.0)	10 (22.2)	0 (0.0)		10 (19.6)	
T3	10 (20.0)	6 (13.3)	1 (16.7)		7 (13.7)	
T4	23 (46.0)	24 (53.3)	3 (50.0)		27 (52.9)	

*Nodal status*^a^						
N0	19 (38.0)	28 (62.2)	3 (50.0)	0.062	31 (60.8)	0.022
N+	31 (62.0)	17 (37.8)	3 (50.0)		20 (39.2)	

*Histopathological grade*						
Well	10 (20.0)	15 (33.3)	0 (0.0)	0.192	15 (29.4)	0.238
Moderately	12 (24.0)	14 (31.1)	3 (50.0)		17 (33.3)	
Poorly	4 (8.0)	1 (2.2)	0 (0.0)		1 (2.0)	
Not available b	24 (48.0)	15 (33.3)	3 (50.0)		18 (35.3)	

Features	*MTHFR* A1298C genotype					
	AA	AC	CC	*p*-value	AC + CC	*p*-value
	*n* (%)	*n* (%)	*n* (%)		*n* (%)	
*Tumor stage*						
Early stage (I, II)	14 (23.3)	10 (27.8)	1 (20.0)	0.860	11 (26.8)	0.689
Advanced (III, IV)	46 (76.7)	26 (72.2)	4 (80.0)		30 (73.2)	

*Tumor size*^a^						
T1	6 (10.0)	6 (16.7)	0 (0.0)	0.747	6 (14.6)	0.512
T2	16 (26.7)	5 (13.9)	1 (20.0)		6 (14.6)	
T3	10 (16.7)	6 (16.7)	1 (20.0)		7 (17.1)	
T4	28 (46.7)	19 (52.8)	3 (60.0)		22 (53.7)	

*Nodal status*^a^						
N0	30 (50.0)	19 (52.8)	1 (20.0)	0.386	20 (48.8)	0.904
N+	30 (50.0)	17 (47.2)	4 (80.0)		21 (51.2)	

*Histopathological grade*						
Well	8 (13.3)	14 (38.9)	3 (60.0)	0.037	17 (41.5)	0.007
Moderately	18 (30.0)	10 (27.8)	1 (20.0)		11 (26.8)	
Poorly	5 (8.3)	0 (0.0)	0 (0.0)		0 (0.0)	
Not available^b^	29 (48.3)	12 (33.3)	1 (20.0)		13 (31.7)	

a TNM classification.

b Not available (not considered in the statistical calculations).

**Table 6:** Clinicopathological characteristics of patients with OSCC and relation with the *CBS* polymorphism studied.

Features	*CBS* 844ins68 genotype		
	Non-insertion	Insertion	*p*-value
	*n* (%)	*n* (%)	
*Tumor stage*			
Early stage (I, II)	18 (23.7)	7 (28.0)	0.664
Advanced (III, IV)	58 (76.3)	18 (72.0)	

*Tumor size*^a^			
T1	8 (10.5)	4 (16.0)	0.792
T2	18 (23.7)	4 (16.0)	
T3	13 (17.1)	4 (16.0)	
T4	37 (48.7)	13 (52.0)	

*Nodal status*^a^			
N0	40 (52.6)	10 (40.0)	0.273
N+	36 (47.4)	15 (60.0)	

*Histopathological grade*			
Well	19 (25.0)	6 (24.0)	0.462
Moderately	22 (28.9)	7 (28.0)	
Poorly	5 (6.6)	0 (0.0)	
Not available^b^	30 (39.5)	12 (48.0)	

a TNM classification.

b Not available (not considered in the statistical calculations).

**Table 7:** Multivariate analysis of the nodal status, according to tumor size and *MTHFR* C677T polymorphism.

Nodal status (N)^a^		
Variable	Multivariate analysis	
	OR (95% CI)	*p*-value
*Tumor size (T)*^a^		
T1, T2	1	
T3	3.07 (0.87–10.87)	0.083
T4	6.46 (2.32–17.95)	<0.001

*MTHFR* C677T		
CC	1	
CT + TT	0.32 (0.13–0.77)	0.012

OR, odds ratio; CI, confidence interval.

a TNM classification.

We observed that the A1298C polymorphism is related to tumor differentiation. AC + CC genotypes were more frequent in well-differentiated tumors, whereas the AA genotype was more frequent in moderately or poorly differentiated tumors (*p* = 0.007) (Table 5).

There was no statistically significant association between *CBS* 844ins68 polymorphism and analyzed variables (Table 6).

## Discussion

Folate deficiency has been associated with diseases such as cancer. Therefore, the role of genetic polymorphisms of folate metabolism enzymes has been investigated in several cancer types.^17^, ^18^ Among these enzymes, we investigated *MTHFR* and *CBS* in OSCC.

Our study has reported for the first time the association between lymph node metastasis and *MTHFR* C677T polymorphism combination CT + TT, acting as a protective factor (*p* = 0.022). Furthermore, other studies have suggested a better prognosis for oral cancer patients with CT or TT genotypes. Tsai et al.^19^ showed that 677CT + TT patients had a lower risk of metastasis compared with those with CC, and Sailasree et al.^20^ showed improved survival.

The protective effect could be due to its decreased efficiency for DNA methylation.^21^ *MTHFR* converts 5,10-MTHF to 5-MTHF. The 5,10-MTHF is used for conversion of dUMP to dTMP, whereas 5-MTHF is the methyl donor for synthesis of methionine and S-adenosylmethionine in methylation reactions.^22^ Individuals with *MTHFR* 677TT and *MTHFR* 677CT genotypes show enzymes with decreased activity,^8^ hence they tend to accumulate 5,10-MTHF causing a change in the pathway, leading to lowering of DNA methylation.^21^ A low level of genomic DNA methylation could decrease the chance of promoter hypermethylation in cancer-related genes and lead to fewer mutations by spontaneous deamination of 5-methylcytosine (5mC). Hypermethylation favors cancer initiation and progression by silencing tumor suppressor genes or DNA repair genes.^23^ Most mutations found in cancers are C → T transitions at CG:CG sequences, due to high frequencies of spontaneous deamination of 5 mC.^24^, ^25^ Lack of cytosine methylation could prevent C → T mutations.

In addition, the *MTHFR* A1298C polymorphism was associated with tumor differentiation. AC + CC genotypes were more frequent in well-differentiated tumors (*p* = 0.007), which may also be associated with a better prognosis, while the AA genotype had a higher frequency in moderately or poorly differentiated tumors. Although the prognostic value of the histological grade is controversial in OSCC, some studies have suggested that poorly differentiated carcinomas tend to metastasize and are associated with decreased survival rates.^26^ However, there is no consensus in the literature about whether histological tumor grading is a good isolated parameter of prognosis value. Tumor histopathological grade, together with additional prognostic factors and TNM staging might provide better support for treatment decision.

However, in this study with a southeastern Brazilian population, we did not observe association between *MTHFR* C677T, *MTHFR* A1298C and *CBS* 844ins68 polymorphisms and oral cancer susceptibility. Some studies have also found a non-significant reduced risk for *MTHFR* 677TT genotype in oral cancer.^27^, ^28^ In contrast, Sailasree et al.^20^ had found that C677T was associated with predisposition to oral cancer with a significant reduced risk for CT + TT genotype individuals. Meta-analysis studies^29^, ^30^ have shown a marginal association or no associations of *MTHFR* C677T polymorphism with oral cancer risk. The conflicting results regarding the associations between *MTHFR* C677T polymorphisms and risks for OSCC may be due to different ethnicities, subtypes and regional dietary and local carcinogens’ exposure.

For A1298C polymorphism, some studies agree with our results, including a meta-analysis study^19^, ^20^, ^31^ showing a lack of association with oral cancer risk. However, meta-analysis demonstrated that C allele has a possible preventive role for oral cancer.^29^

In our study, the CBS 68 bp insertion allele (I) was neither associated with OSCC risk nor with heterozygous genotype (I/N) or polymorphic homozygous genotype (I/I), corroborating the results of Galbiatti et al.,^32^ but unlike results for other tumor types such as prostate and upper gastrointestinal tract cancers.^33^, ^34^

*MTHFR* C677T polymorphism genotype prevalence varies to a great extent among different human populations. In Indians, the frequency of TT genotype is below 1 percent,^20^ whereas among Mexicans, it is above 30 percent; the TT genotype in our controls showed a 10.8 percent frequency, which is comparable to previous reports from Chinese and Puerto Rican populations,^27^, ^35^, ^36^, ^37^ and also in regions of Brazil.^38^, ^39^, ^40^ The frequencies of TT genotype demonstrated in studies with populations in southeastern Brazil range from 4.4 percent to 14 percent in the state of São Paulo.^39^, ^41^, ^42^ Control prevalence of variant *MTHFR* A1298C genotypes (CC) in our study was 4.9 percent, in agreement with studies in northeastern Brazil and also in other populations of the world, such as Chinese, Japanese, Polish, Italian and Americans^19^, ^43^, ^44^, ^45^, ^46^, ^47^, ^48^; however, there was little difference from frequencies observed in the state of São Paulo, with frequencies of 6.1 percent and 8.8 percent.^41^, ^42^

In our study, the *CBS* 68 bp insertion allele (I) was found in 7.8 percent of the control population. A similar frequency (7%) was observed in the Pakistani population^49^; however, the allelic frequency was higher in another study conducted in São Paulo, Brazil.^32^ As found in another study,^32^ *CBS* 844ins68 polymorphism was not associated with clinical or histopathological features in our study.

## Conclusion

In conclusion, *MTHFR* C677T CT and TT genotypes were associated with lymph node involvement, acting as a protective factor in OSCC, and the *MTHFR* A1298C AC + CC genotype was associated with tumor differentiation, which may be associated with a better prognosis. However, the results need to be confirmed in larger studies of patients and controls matched by smoking habit.

## Conflicts of interest

The authors declare no conflicts of interest.
